# Correction: Factors Limiting Performance in a Multitone Intensity-Discrimination Task: Disentangling Non-Optimal Decision Weights and Increased Internal Noise

**DOI:** 10.1371/journal.pone.0097209

**Published:** 2014-05-02

**Authors:** The PLOS ONE Staff

## Notice of Republication

This article was republished on April 14, 2014 to replace an incorrectly published version where Equations 10 and 11 were typeset incorrectly. Please download this article again to view the correct version. The originally published, uncorrected article and the republished, corrected article are provided here for reference.

## Supporting Information

File S1 Originally published, uncorrected article.
(PDF)

File S2 Republished corrected article.
(PDF)
