# Supplementary material for: Correction: Factors Limiting Performance in a Multitone Intensity-Discrimination Task: Disentangling Non-Optimal Decision Weights and Increased Internal Noise
Source: PLoS One. 2014 May 2;9(5):e97209. doi: 10.1371/journal.pone.0097209 (PMC4008626; doi:10.1371/journal.pone.0097209)
Supplement: File S2 — Republished corrected article. (PDF) [file pone.0097209.s002.pdf]

PLOS ONE

Factors Limiting Performance in a Multitone Intensity-Discrimination Task: Disentangling Non-Optimal Decision Weights and Increased Internal Noise

Daniel Oberfeld[1]*, Martha Kuta[1], Walt Jesteadt[2]

1 Department of Psychology, Johannes Gutenberg-Universität Mainz, Mainz, Germany, 2 Psychoacoustics Laboratory, Boys Town National Research Hospital, Omaha, Nebraska, United States of America

Abstract

To identify factors limiting performance in multitone intensity discrimination, we presented sequences of five pure tones alternating in level between loud (85 dB SPL) and soft (30, 55, or 80 dB SPL). In the ''overall-intensity task'', listeners detected a level increment on all of the five tones. In the ''masking task'', the level increment was imposed only on the soft tones, rendering the soft tones targets and loud tones task-irrelevant maskers. Decision weights quantifying the importance of the five tone levels for the decision were estimated using methods of molecular psychophysics. Compatible with previous studies, listeners placed higher weights on the loud tones than on the soft tones in the overall-intensity condition. In the masking task, the decisions were systematically influenced by the to-be-ignored loud tones (maskers). Using a maximum-likelihood technique, we estimated the internal noise variance and tested whether the internal noise was higher in the alternating-level five-tone sequences than in sequences presenting only the soft or only the loud tones. For the overall-intensity task, we found no evidence for increased internal noise, but listeners applied suboptimal decision weights. These results are compatible with the hypothesis that the presence of the loud tones does not impair the precision of the representation of the intensity of the soft tones available at the decision stage, but that this information is not used in an optimal fashion due to a difficulty in attending to the soft tones. For the masking task, in some cases our data indicated an increase in internal noise. Additionally, listeners applied suboptimal decision weights. The maximum-likelihood analyses we developed should also be useful for other tasks or other sensory modalities.

Citation: Oberfeld D, Kuta M, Jesteadt W (2013) Factors Limiting Performance in a Multitone Intensity-Discrimination Task: Disentangling Non-Optimal Decision Weights and Increased Internal Noise. PLoS ONE 8(11): e79830. doi:10.1371/journal.pone.0079830

Editor: Warren H. Meck, Duke University, United States of America

Received May 17, 2013; **Accepted** October 4, 2013; **Published** November 20, 2013

Copyright: © 2013 Oberfeld et al. This is an open-access article distributed under the terms of the Creative Commons Attribution License, which permits unrestricted use, distribution, and reproduction in any medium, provided the original author and source are credited.

Funding: This work was supported by a grant from Deutsche Forschungsgemeinschaft (DFG) to Daniel Oberfeld (OB 346/4-1: Temporal aspects of auditory intensity processing). The funders had no role in study design, data collection and analysis, decision to publish, or preparation of the manuscript. No additional external funding was received.

Competing Interests: The authors have declared that no competing interests exist.

* E-mail: oberfeld@uni-mainz.de

Introduction

In experiments on multitone intensity discrimination (also termed multitone pattern discrimination), the stimulus consists of several sequentially presented elements and the task is to judge the intensity of this multitone stimulus (e.g., [1,2]). The focus of most of these experiments has been on estimating the relative reliance or decision weight listeners give to different tones in the pattern, by using so-called *molecular psychophysical analyses* (cf. [3,4,5,6]), also known as *perceptual weight analysis* or *psychophysical reverse correlation*.

Previous research showed that listeners do not make optimal use of the intensity information available in a multitone pattern when the tones differ in sound pressure level [7]. The aim of this study was to identify sensory and decisional factors contributing to this behavior. Lutfi and Jesteadt [7] conducted a multitone intensity discrimination experiment where each stimulus consisted of five sequentially presented tones. The tones alternated in level, so that for example the mean level of the first tone was 80 dB SPL, the mean level of the second tone was 40 dB SPL, and the mean level of the third tone was again 80 dB SPL (see Fig. 1 for a similar stimulus configuration). The task of the listener was to detect a level increment imposed on all of the five tones. The levels of the five tones were randomly perturbed, and the random perturbation was used to estimate decision weights for each of the five tones (cf. [4,6]). The listeners placed much higher weight on the loud tones than on the soft tones, even if the level information from the soft tones was rendered more reliable by presenting a larger level increment on the soft than on the loud tones. This pattern of weights is consistent with other reports of a ''level dominance'' effect [8,9]. For instance, it has been demonstrated that the temporal level profile of longer, level-fluctuating sounds strongly influences the pattern of perceptual weights assigned to individual temporal portions of the sounds when listeners judge the overall intensity. For sounds beginning with a gradual increase in level, the weights assigned to the attenuated (''fade in'') part are close to zero [2]. For sounds beginning with a gradual decrease in level, on the other hand, the first part of the sound receives the highest weight [5]. Both observations are compatible with attention to the loudest elements [8]. Notably, in an additional experiment by Lutfi and Jesteadt [7] where the loud elements were wideband noise bursts rather than pure tones, listeners placed the highest weights on the more reliable soft tones. Lutfi and Jesteadt interpreted these data as to

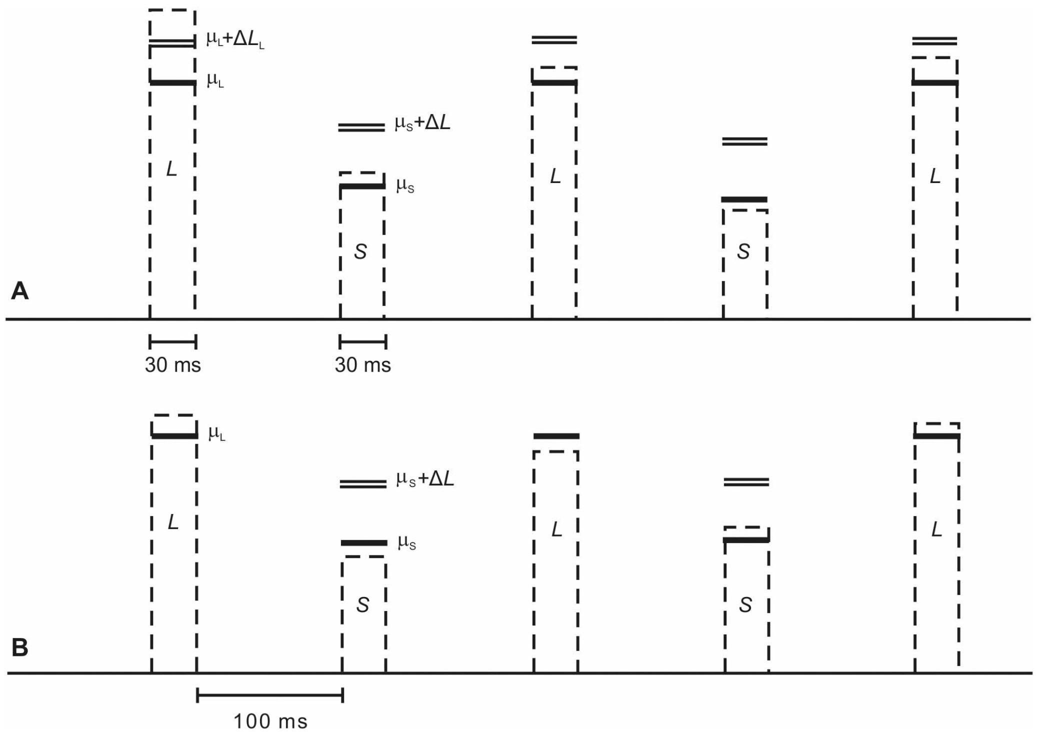

Figure 1. Trial configuration in the multitone intensity-discrimination task with alternating loud and soft tones. Panel A shows the *overall-intensity task*. Each trial contained three loud tones (*L*) and two soft tones (*S*). The level of each tone was sampled independently from a normal distribution. Mean levels (μ) are represented by the thick black lines. The actual levels presented in the example trial are shown by the dashed lines. In the example, a level increment of 4 dB (ΔL_L) is added to the loud tones and a level increment of 6 dB (ΔL_S) is added to the soft tones, indicated by the double line. The task was to decide whether the overall loudness of the trial was loud (i.e., contained the increments) or soft (i.e., did not contain the increments). Panel B shows the *masking task* in which the level increments were presented only on the soft tones (i.e., targets), and the loud tones were to be ignored (i.e., maskers). The task was to decide whether the two soft tones (targets) contained the increments. Listeners were instructed to ignore the loud tones (maskers).
doi:10.1371/journal.pone.0079830.g001

showing that the spectral difference between soft and loud sounds facilitated the direction of attention to the soft elements.

Do the different weights assigned to the soft and the loud tones in the multitone stimulus thus represent a purely "cognitive" effect in the sense of a suboptimal decision strategy? The motivation for the present study was that there is an alternative explanation, and our aim was to decide between the two explanations. Lutfi and Jesteadt [7] assumed that the intensity resolution for the soft and the loud tones did not differ dramatically. More specifically, they assumed the intensity resolution for each tone embedded in the multitone sequence to be identical to intensity resolution in quiet, where the near-miss to Weber's law [10] describes the slightly higher intensity resolution for tones with a higher sound pressure level. However, the intense tones could have acted as non-simultaneous maskers for the softer tones. As described above, in one condition studied by Lutfi and Jesteadt, the mean level of the loud tones was 80 dB SPL, the mean level of the soft tones was 40 dB SPL, and the ISI between for example the first (loud) tone and the second (soft) tone was 100 ms (cf. Fig. 1). This configuration is similar to the stimuli presented in a forward-masked intensity-discrimination task (see Fig. 1 in [11]), where a strong masker-induced elevation of the intensity difference limens for the 40 dB SPL tones would be expected if combined with an 80 dB SPL masker at the described temporal configuration [12]. Thus, if non-simultaneous masking played a role, then the intensity difference limen (DL) for the 40 dB SPL tone might have been 5–20 dB higher than for the 80 dB SPL tone (e.g., [11]). A strong DL elevation would have rendered the level increment of 6 dB placed on the soft tones in the experiment by Lutfi and Jesteadt virtually undetectable. In such a case, the optimal strategy would be to place only small weight on the soft tones. Put differently, the listener should rely mainly on level information from the loud tones, even if he or she was in principle able to attend to the soft tones. The observation that the soft tones received higher weight if the loud tones were replaced by noise bursts at the same sound pressure level is also compatible with an explanation based on intensity resolution because the wideband noise maskers contained much less energy in the auditory filter centered at the signal frequency, and can therefore be assumed to have had a smaller impact on intensity resolution for the target tones [12].

On a more general level, both *sensory* and *decisional* (cognitive) factors may have contributed to the weighting pattern observed by Lutfi and Jesteadt [7] in the multitone paradigm. We conceptualize the performance in the multitone intensity discrimination task in terms of a very simple processing model. In the first stage (sensory processing), each tone is processed by the auditory system, resulting in a representation of the intensity of each tone. These representations are stored in a memory system. The second stage (decision stage) then combines the level representations and outputs a decision according to some decision rule. For reasons of simplicity, we conceive the combination of information to be lossless, i.e., in the signal detection theory tradition there is no decision noise. Now, the representation of the intensity of the soft tones available at the decision stage could be impaired by the presence of the loud

tones (i.e., temporal masking), for example due to adaptation in the auditory nerve [13]. This loss of information in the first stage of our simple model can be described as an increase in *internal noise* [14], and for the sake of simplicity we make no attempt to distinguish between sensory noise and memory noise [15]. The higher weights assigned to the loud tones could then be regarded as optimal because the information about the intensity of the loud tones available at the decision stage is more reliable than the information about the intensity of the soft tones [16]. Alternatively, the representation of soft-tone intensity available at the decision stage could be unaffected by the loud tones (i.e., no increase in internal noise) but the listeners could assign *non-optimal decision weights*. The latter explanation would be in line with the attention hypothesis put forward by Lutfi and Jesteadt [7]. To determine which of the two factors play a role in the multitone intensity-discrimination task, we estimated the internal noise in the different conditions via a novel type of maximum-likelihood analysis.

Given the background of previous research, we expected suboptimal decision strategies to play a more important role than increases in internal noise. First, in multitone discrimination tasks involving judgments of frequency rather than intensity, effects of relative tone level have been observed at ISIs up to 500 ms [8,9]. At this temporal separation, effects of forward masking on frequency discrimination should be negligible [17], although backward masking was reported to be present for intervals up to 240 ms [18,19]. Thus, previous studies suggest that the effect of relative tone level on the decision weights is at least partly caused by factors other than non-simultaneous masking. However, for intensity discrimination, effects of non-simultaneous maskers have been reported at considerably longer masker-target ISIs than in frequency discrimination [12], and backward maskers cause similar effects as forward maskers [20,21,22]. Specifically, in the stimuli presented by Lutfi and Jesteadt [7], the temporal separation between adjacent tones was just within the range for which strong effects of non-simultaneous masking were reported in an intensity discrimination task [12,20]. Thus, temporal masking may have played a role here.

However, this is not to imply that decisional factors play no role in experiments on intensity discrimination under non-simultaneous masking. Here, an important finding is the so-called *mid-level hump* [13]. The mid-level hump refers to the non-linear relationship between the level of the standard and the elevation of the intensity DL caused by an intense non-simultaneous masker (e.g. 90 dB SPL). The DL-elevation is more pronounced for a mid-level standard than for standards low or high in level. This result has been explained by several authors with a focus on reduced precision of the information about target intensity, either at the level of the auditory nerve or at later processing stages (for reviews see [11,23]). Thus, in terms of our simple model, the maskers were assumed to increase the internal noise present at stage 1. However, previous studies from our lab showed that the failure of selective attention to the targets is a useful framework for understanding the effects of non-simultaneous masking on intensity resolution [11,22,23,24,25]. This argues for suboptimal information integration in the decision stage. The hypothesis that the excessive weight placed on the loud tones in the multitone intensity discrimination task is largely due to non-optimal weighting strategies is in line with this concept.

Thus, in terms of both processing stages of our simple model, the performance in a multitone intensity discrimination task involving a sequence of tones alternating in level might be related to classical forward-masked intensity discrimination. For this reason, we expected a mid-level hump pattern [13] in the multitone paradigm, which would be a qualitative indication of the similarity between listeners' performance in the two tasks. We presented three different combinations of the level of the loud and soft tones. Our hypothesis was that if the mean level of the loud tones is fixed at 85 dB SPL, then the decision weights assigned of the soft tones will be even lower if they are presented at 55 dB SPL than if they are presented at 30 dB SPL.

Methods

To estimate the decision weights assigned to the different tones in the multitone stimulus, a one-interval, absolute identification task [26] was used. As depicted in Fig. 1, each trial included five tones alternating in mean level as in the experiments by Lutfi and Jesteadt [7,27]. The levels of all tones were randomly perturbed. The mean level of the loud tones was fixed at 85 dB SPL. The mean level of the soft tones was either 30 dB SPL, 55 dB SPL, or 80 dB SPL, resulting in masker-target level combinations like those in studies of the mid-level hump [13].

Each combination of the level of the soft and the loud tones was studied in two conditions. In one condition, a level increment was placed on all tones, just as in the experiments by Lutfi and Jesteadt [7]. Therefore, the task could be described as a global loudness judgment [28] where listeners judged the loudness of the stimulus in its entirety (i.e., over all five tones). In the other condition, the increment was placed only on the soft tones. As a consequence, like in a typical forward-masked intensity-discrimination task (e.g., [13]) the soft tones were "targets" and the remaining tones were task-irrelevant "maskers". In the latter condition, the listeners were instructed to judge the intensity of the soft tones and to ignore the loud tones.

Ethics Statement

The experiment was conducted according to the principles expressed in the Declaration of Helsinki. All listeners participated voluntarily after providing informed written consent, after the topic of the study and potential risks had been explained to them. They were uninformed about the experimental hypotheses. The study was approved by the ethical review board of the Department of Psychology at the Johannes Gutenberg-Universität Mainz.

Participants

Seven students at the Johannes Gutenberg-Universität Mainz participated in the experiment voluntarily (6 female, 1 male; aged 21–30 years). They either received partial course credit or were paid for their participation. All listeners reported normal hearing. Detection thresholds measured by Békésy tracking [29,30] with pulsed 270-ms tones including 10-ms $\cos^2$ on- and off-ramps were better than 20 dB HL between 125 Hz and 8 kHz. Listeners were screened for acceptable detection thresholds under forward masking (see section *Screening* below).

Furthermore, they were screened for showing a mid-level hump in an intensity-discrimination task with only one forward masker. It is known from previous experiments that for some subjects the masker-induced DL elevation is maximal at the lowest rather than at an intermediate target level (for a discussion see [23]). In terms of an ideal observer argument, for these listeners the weights should increase monotonically as a function of mean presentation level. As a consequence they cannot be used to test the hypothesis of this study pertaining to the mid-level hump.

Stimuli and Apparatus

The multitone stimulus consisted of 1000 Hz pure tones presented to the right ear, with a steady-state duration of 20 ms, gated on and off with 5-ms cosine-squared ramps. Each sinusoid started at zero phase. The multitone stimulus comprised a sequence of five tones separated by silent intervals of 100 ms, measured between zero-voltage points (see Fig. 1). The tones alternated in mean level, with the second and fourth tone having softer mean levels ("soft tones") than the other three tones ("loud tones").

In the *overall-intensity task*, a level increment – that is, a pure tone of the same frequency, duration and temporal envelope – was added in-phase to each of the five tones. In the *masking task*, increments were added only to the soft tones, rendering them "targets" because the loud tones ("maskers") no longer provided any task-relevant information. The sound pressure levels of the loud tones were drawn independently from a uniform distribution spanning a symmetric range of $q = 12$ dB, centered symmetrically on the mean level $\mu_{\mathrm{loud}} = 85$ dB SPL. This distribution has a standard deviation of $\sigma = \sqrt{1/12 \cdot q^2} = 3.46$ dB [31]. The soft tones were drawn independently from a uniform distribution with mean $\mu_{\mathrm{soft}} = 30$ dB SPL, 55 dB SPL, or 80 dB SPL, again with a range of 12 dB.

To account for the near-miss to Weber's law (cf. [7,10]), that is, lower intensity resolution at low compared to high target levels, we adopted level increments of $\Delta L_{\mathrm{L}} = 4$ dB for the loud tones and $\Delta L_{\mathrm{S}} = 6$ dB for the soft tones. The inter-trial interval was 2000 ms, with the restriction that the next trial never started before the response and the feedback to the preceding trial had been given. A trial started with a 300-ms visual attention signal provided by two LEDs, followed by a silent interval of 500 ms, and then the onset of the first tone. The LEDs also visually marked the task-relevant target tones, and were used to provide trial-by-trial feedback.

The stimuli were generated digitally, played back via one channel of an RME ADI/S D/A converter ($f_{\mathrm{s}} = 44.1$ kHz, 24-bit resolution), attenuated by a TDT PA5 programmable attenuator, buffered by a TDT HB7 headphone buffer, and presented to the right ear via Sennheiser HDA 200 circumaural headphones calibrated according to IEC 318 [32]. The experiment was conducted in a double-walled sound-insulated chamber.

Procedure and Design

Multitone intensity discrimination with alternating loud and soft tones. For the multitone sequence of five tones alternating in level, two different tasks were presented. In the *overall-intensity task*, level increments ΔL were added to each of the five tones; in the *masking task* they were added only to the soft tones. Each trial had an a priori probability of 50% to contain the increments. Listeners were instructed to indicate if the just presented multitone stimulus had been soft or loud compared to previous trials in a given block. Thus, an absolute identification task [26] with a virtual standard (e.g., [33]) was used. One could also describe the task as a sample discrimination task [1,34,35] where the listeners decided whether the tone levels had been drawn from the "loud distribution" (i.e., increments present) or the "soft distribution" (increments absent). In the *overall-intensity task*, to emphasize the necessity to judge the overall loudness of the trial, LED 1 was switched at the onset of the first tone, and was switched off at the offset of the fifth tone. In the *masking task*, the task was to judge the intensity of only the two soft tones ("targets"), and to ignore the loud tones ("maskers"). LED 1 was switched on in synchrony with the targets. Visual trial-by-trial feedback was provided. Subjects were informed that due to the random level perturbations the feedback could be counterintuitive on some trials.

Apart from the decision weights, we were interested in the sensitivity for detecting the increment. If binary responses (e.g., "soft" versus "loud") are obtained in a one-interval task, then d' can only be estimated from the hit and false-alarm rates under the assumption of equal-variance Gaussian distributions for "signal" and "noise" on the internal continuum (cf. [36,37]). Therefore, a rating response was used and the area under the ROC curve (AUC) was computed, which is a valid measure for sensitivity that does not require strong assumptions about the internal distributions (e.g., [38,39]). AUC corresponds to the proportion of correct responses obtained with the same stimuli in a forced-choice task [36,40,41,42]. The rating scale comprised four ordered response categories ("soft – rather sure", "soft – rather unsure", "loud – rather unsure", "loud – rather sure"). Sensitivity in terms of the AUC was computed for each experimental block (105 trials, first five trials excluded from the analysis) and then transformed to d', using the relation $d'_{2\mathrm{I}} = \sqrt{2}z(\mathrm{AUC})$, where $z(\mathrm{AUC})$ is the standard normal deviate corresponding to the proportion AUC (cf. [38,40,41,43]). The advantage of using d' rather than AUC is that d' can be viewed as a linearization of the binomial quantity AUC, and d' is often found to be linearly related to stimulus magnitude (e.g., [44,45]). A linearization is also desirable because repeated-measures ANOVAs are sensitive to departures from normality [46].

For each combination of task (*overall-intensity task*, *masking task*) and mean level of the soft tones ($\mu_{\mathrm{soft}} = 30$, 55, 80 dB SPL), five blocks of 105 trials and thus a total of 525 trials were presented. The first five trials of each presentation block were removed from further calculations, reflecting the necessity to first establish a decision criterion at the beginning of an experiment block. Therefore, 500 trials per listener and condition entered the analysis. Only one of the two tasks was presented during one session, in order to avoid confusion between the two different tasks. Only one value of μ_{soft} was presented in each block. The order of blocks was randomized in each session.

To obtain information about intensity resolution for only the soft or only the loud tones, in four additional conditions only the two soft tones (30, 55, or 80 dB SPL), or only the three loud tones (85 dB SPL) were presented. These stimuli were generated simply by deleting the loud or the soft tones, respectively, from the five-tone sequences. Thus, in the *soft-tones-only* and *loud-tones-only* tasks, all tones were presented at the same temporal position as in the five-tone sequences. The same random level perturbations and level increments as in the five-tone sequences were used. For each of the four different mean levels, 420 trials were collected (also omitting the first five trials per block, i.e. 400 trials were entered into the data analyses).

Estimation of decision weights. The individual trial-by-trial data obtained for each combination of task (overall-intensity, masking, soft-tones/loud-tones-only) and mean level of the soft tones were analyzed to estimate the decision weights. Multiple logistic regression (PROC LOGISTIC of SAS 9.2) was used to estimate the influence of the level of each individual tone on the response of the listener (cf. [2,27,28,47]). The rating of perceived target loudness ("soft – rather sure", "soft – rather unsure", "loud – rather unsure", and "loud – rather sure") served as the dependent variable and the two to five tone levels served as predictors, which were entered simultaneously. The regression coefficients were taken as the weight estimates. For a given tone, a regression coefficient equal to zero means that the level of the tone had no influence at all on the decision to judge the target tones as being either soft or loud. A regression coefficient greater than zero

means that the probability of responding that the target tones of the presented sequence were perceived as loud rather than soft increased with the level of the given tone. A regression coefficient less than zero indicates the opposite relation between the level of one tone and the probability of responding that the targeted tones were perceived as loud rather than soft.

This analysis is based on a decision model which assumes that listeners use a decision variable.

$$D_j(\mathbf{L}) = \left(\sum_{i=1}^{k} w_i L_i\right) - c_j, \qquad (1)$$

where L_i is the sound pressure level of tone i, k is number of tones, $\mathbf{L}$ is the vector of tone levels, w_i is the perceptual weight assigned to tone i, and c_j is a constant representing the decision criterion for the jth of the four ordered response categories (cf. [6,28,48]). In other words, $D_j(\mathbf{L})$ is a weighted average of the different sound pressure levels of tones. As we have a four-category response variable Y we assumed a proportional-odds model [49] according to which

$$P(Y \leq j) = \frac{e^{D_j(\mathbf{L})}}{1 + e^{D_j(\mathbf{L})}},\ j = 1,\ \ldots, J-1, \qquad (2)$$

where J is the number of ordered response categories. This model applies simultaneously to all $J-1$ cumulative probabilities, and it assumes an identical effect of the predictors for each cumulative probability [48].

Due to the difference in mean level between "increment present" and "increment absent" trials, the tone levels were correlated. Although multiple logistic regression should correct for these correlations, to avoid any problems with multicollinearity separate logistic regression analyses were conducted for the trials containing increments vs. no added increments (cf. [6]). Thus, a separate logistic regression model was fitted for each combination of subject, mean level of soft (loud) tones, task and increment (present/absent). As we were interested in the relative contributions of the different tones levels to the decision rather than in the absolute magnitude of the regression coefficients, the weights w_i were normalized such that the sum of the absolute values of the weights was unity (see [5]), resulting in a set of relative temporal weights for each fitted model.

Detection thresholds. Detection thresholds under masking and in quiet were obtained in a 2I, 2AFC task. The second and fourth tones of the multitone sequence (i.e., the soft tones) were presented in one of the two observation intervals. The target tones occurred with equal probability in the first or second interval. In the detection task under masking, three maskers corresponding to the first, third and fifth tone of the multitone sequence were presented at the maximal masker level from the discrimination task (95 dB SPL). This level corresponds to a mean tone level of 85 dB SPL, a level perturbation of +6 dB, and a 4 dB level increment. No random level perturbation was applied, either to the maskers or to the signal. The two observation intervals were separated by a silent interval of 600 ms. An adaptive procedure with a 3-down, 1-up tracking rule was used [50]. Initially, the signal level was 40 dB SPL. The step size was 8 dB until the fourth reversal, and 2 dB for the remaining eight reversals. A track ended when 12 reversals had been obtained or when 70 trials had been presented, whichever occurred first. Visual trial-by-trial feedback was provided. The threshold level was computed as the arithmetic mean of the signal levels at the final eight reversals. A track was discarded if the standard deviation of the latter signal levels was greater than 6 dB. For both detection under masking and detection in quiet, four adaptive blocks were obtained.

Screening. To screen for an acceptable threshold under masking, forward-masked detection thresholds were measured prior to the main experiment. Again, the masker level was 95 dB SPL. The criterion for participation in the main experiment was a threshold of 19 dB SPL or lower. Because the minimum target level was 24 dB SPL in the main experiment, this ensured that the target tones were at least 5 dB above the detection threshold in all trials of the main experiment. Three adaptive blocks were obtained, using the procedure described above.

To screen for a mid-level hump in a typical forward-masked intensity-discrimination task, prior to the main experiment intensity DLs were measured, using the same one-interval task as in the main experiment but presenting only one forward masker and one target. The same frequency, tone duration, and masker-target ISI as in the multitone task were used. The same random level perturbations as in the multitone tasks were applied to the target. No random level perturbation was applied to the masker. A fixed level increment was added to the target, no increment was added on the masker. The listeners were instructed to ignore the masker and to judge whether the target was loud or soft, using the rating scale introduced above. Visual trial-by-trial feedback was provided. A forward masker with a level of 85 dB SPL combined with either a mean target level of 30 or 55 dB SPL was presented. Although the definition of the mid-level hump implies both a lower and upper comparison, we did not include a high-level target to save experimentation time because without exception all previous studies showed very small DL-elevations at high target levels. Cumulative-normal psychometric functions relating the (randomly varying) sound pressure level of the target to the proportion of trials on which the listener responded that the loud target had been presented [51] were fitted for each block, using a maximum-likelihood method. The DL was defined as half the difference between the 75%-point and the 25%-point on the psychometric function.

For each listener and for each mean target level 420 trials were obtained. Again, the first five trials of each block were excluded from further analyses, i.e. 400 trials were used. Listeners were included into the main experiment if they showed an at least 4 dB higher DL for the mean target level of 55 dB SPL than for the mean target level of 30 dB SPL. This resulted in the exclusion of 4 out of a total of 11 listeners. Averaged across the listeners selected for the experiment, the difference between the DL at the 55 dB SPL target level ($M = 25.67$ dB, $SD = 8.70$ dB) and the DL at the 30 dB SPL target level ($M = 12.86$ dB, $SD = 3.94$ dB) was 12.81 dB ($SD = 5.9$ dB), showing a substantial mid-level hump.

Sessions. Each listener participated in a total of eleven experimental sessions, each with a duration of approximately 55 minutes. The first two sessions served as screening sessions for acceptable thresholds under masking as well as for showing a mid-level hump pattern in the individual data under forward masking. Audiometric thresholds were measured in session 1. In session 3, practice blocks for all tasks were presented. In the remaining eight sessions constituting the main experiment, two alternating configurations were presented. The even-numbered sessions presented four blocks of the masking task and two blocks of the soft−/loud-tones-only conditions. Each block contained 105 trials. The order of tasks was randomly selected. The four blocks of the masking condition were randomly selected from the set of three mean levels of the soft tones. The two blocks of the soft−/loud-tones-only conditions were randomly selected from the four different conditions (soft-tones-only with $\mu_{soft} = 30$, 55, 80 dB SPL, loud-tones-only). The odd-numbered sessions presented four

blocks of the overall-intensity task and two blocks of the soft−/loud-tones-only condition. Again, the task order was randomly selected. Additionally, at the end of sessions 4 to 12, one block of the detection task (in quiet or under forward masking) was presented.

Results

Detection Thresholds

The average detection threshold (corresponding to 79.4% correct) under masking ($M = 12.23$ dB SPL, $SD = 4.25$ dB; maximum individual value 18.3 dB SPL) was significantly higher than in quiet ($M = 8.58$ dB SPL, $SD = 2.37$ dB), $t(6) = 4.07$, $p < .01$, Cohen's [52] $d_z = 1.54$. As the minimum level of the tones in the intensity-discrimination task was 24 dB SPL, the target tones were presented more than 5 dB above threshold on all trials.

Decision Weights

Before reporting the statistical analyses, we describe the pattern of average normalized decision weights for the overall-intensity task and the masking task.

Panel A of Fig. 2 depicts the decision weights for the overall-intensity task. Compatible with the results by Lutfi and Jesteadt [7], the weights assigned to the loud tones were much higher than the weights assigned to the soft tones at the two lower mean levels of the soft tones (μ_{Soft}). As expected, at $\mu_{Soft} = 80$ dB SPL the weights were more equally distributed. However, at $\mu_{Soft} = 30$ dB SPL the weights assigned to the soft tones were not higher than at $\mu_{Soft} = 55$ dB SPL. Thus, no mid-level hump-like weighting pattern existed for the overall-intensity task.

The average normalized weights in the masking task are shown in Panel B of Fig. 2. For all mean levels of the soft tones, the to-be-ignored loud tones received higher weights, but especially so at the intermediate level of the soft tones. Thus, the data of the masking task exhibited the expected mid-level hump type of weighting pattern. The 95% confidence intervals (CIs) indicate higher inter-individual differences in the masking task than in the overall-intensity task.

The normalized decision weights were analyzed with a repeated-measures analysis of variance (rmANOVA) with the within-subjects factors tone number (tone 1 to tone 5), mean level of the soft tones (μ_{soft}), task, and presence of the increment. A univariate approach with Huynh-Feldt [53] correction for the degrees of freedom (df) was used, which shows good control of the Type I error rate for small sample sizes [46]. The df correction factor $\tilde{\varepsilon}$ is reported. Partial η^2 is reported as a measure of association strength [54]. The same type of rmANOVA was used for the analysis of sensitivity and internal noise (see next two sections *Sensitivity* and *Estimates of internal noise*). We used an α-level of.05 for all analyses. Post-hoc pairwise comparisons were conducted using separate paired-samples *t*-tests [55] and Hochberg's [56] sequentially acceptive step-up Bonferroni procedure which controls the familywise Type I error rate.

There was a significant effect of tone number, $F(4, 24) = 13.66$, $p < .001$, $\eta^2_p = .695$, $\tilde{\varepsilon} = .566$, reflecting the weight differences between loud and soft tones in both tasks. The more uniform weighting profile in the masking task compared to the overall-intensity task resulted in a significant interaction between tone number and task, $F(4, 24) = 6.57$, $p = .003$, $\eta^2_p = .52$, $\tilde{\varepsilon} = .768$. The ANOVA also showed a significant interaction between tone number and μ_{soft}, $F(8, 48) = 5.02$, $p < .001$, $\eta^2_p = .46$, $\tilde{\varepsilon} = .958$, because averaged across both tasks the differences in weights between loud and soft tones were especially pronounced for the sequences containing the 55 dB SPL soft tones and less evident for the sequences containing the 80 dB SPL soft tones. No further main effects or interactions were significant (all *p*-values $> .136$).

To further analyze the tone number×μ_{soft} interaction, two separate three-factorial rmANOVAs (tone number×μ_{soft}×presence of increment) were computed, one for each task. (a) For the overall-intensity task, both the effect of μ_{soft}, $F(2, 12) = 10.41$, $p = .006$, $\eta^2_p = .63$, $\tilde{\varepsilon} = .772$, and the effect of tone number, $F(4, 24) = 58.74$, $p < .001$, $\eta^2_p = .91$, $\tilde{\varepsilon} = .371$, were significant. Also, the μ_{soft}×tone number interaction was significant, $F(8, 48) = 12.39$, $p < .001$, $\eta^2_p = .67$, $\tilde{\varepsilon} = .719$. These results reflect the large differences in weights between soft and loud tones in the overall-intensity task and that these differences are smaller for the sequences containing the 80 dB SPL soft tones. Additionally, the interaction between μ_{soft} and presence of increment was significant, $F(2, 12) = 9.31$, $p = .011$. Inspection of the data showed that this effect was caused by spurious negative weights, which resulted in a difference in average weight because the normalization was computed on the basis of the absolute values of the weights.

(b) For the masking task the effect of tone number was marginally significant, $F(4, 24) = 2.74$, $p = .072$, $\eta^2_p = .31$, $\tilde{\varepsilon} = .762$, as well as the interaction between μ_{soft} and tone number, $F(8, 48) = 1.96$, $p = .097$, $\eta^2_p = .25$, $\tilde{\varepsilon} = .756$. Thus, the differences in weight between soft and loud tones were somewhat weaker than in the overall-intensity task. The non-significant effect of $\mu_{soft,}$ $F(2, 12) = 0.16$, $p = .784$, reflected the similar weights for the sequences with 30 and 80 dB SPL soft tones and the generally more uniform weighting profile in this task (as well as the large inter-individual differences).

For additional exploration of the descriptive mid-level hump pattern in the masking task, we averaged the weights across the three loud and the two soft tones. A three-factorial rmANOVA (tone type (loud/soft)×μ_{soft}×presence of increment) showed a marginally significant effect of tone type, $F(1, 6) = 5.75$, $p = .052$, $\eta^2_p = .49$, reflecting the stronger weights on the loud tones independent of μ_{soft}. A significant interaction between tone type and μ_{soft}, $F(1, 12) = 4.53$, $p = .036$, $\eta^2_p = .43$, $\tilde{\varepsilon} = .969$, confirmed the expected larger difference in weights between soft and loud tones for the sequences containing the 55 dB SPL soft tones ($M_D = 0.19$, $SD = 0.13$) and the more uniform pattern of weights (i.e., smaller differences between weights on soft and loud tones) for the sequences containing the 30 dB SPL ($M_D = 0.05$, $SD = 0.18$) and 80 dB SPL soft tones ($M_D = 0.07$, $SD = 0.08$). For each mean level of the soft tones, post-hoc paired-samples *t*-tests were computed between the averaged weights for the soft tones and the averaged weights for the loud tones, resulting in three comparisons. After correction for multiple testing only the comparison at $\mu_{soft} = 55$ dB SPL was significant.

For the loud tones in the overall-intensity task as well as for loud-tones-only condition, a decrease of weighting values from the first to the last tone is visible in Panel A and Panel D of Fig. 2. This primacy-effect-like pattern (e.g., [57]), is compatible with previous results from multitone intensity-discrimination tasks (e.g., [2,5,28,58,59,60,61]). To investigate this temporal weighting pattern, we analyzed the weights assigned to the loud tones in the overall-intensity task and in the loud-tones-only condition. The three weights in the overall-intensity task were normalized such that the sum of the absolute values was unity. A 3×4 rmANOVA with the factors loud tone number (first, third and fifth tone in the sequence) and task (overall-intensity task at $\mu_{soft} = 30$, 55, and 80 dB SPL, and loud-tones-only condition) showed a significant effect of tone number, $F(2, 12) = 8.34$, $p = .019$, $\eta^2_p = .58$, $\tilde{\varepsilon} = .61$, reflecting the monotonic decrease of the weights from the first, over the third, to the fifth tone. Due to the normalization there was

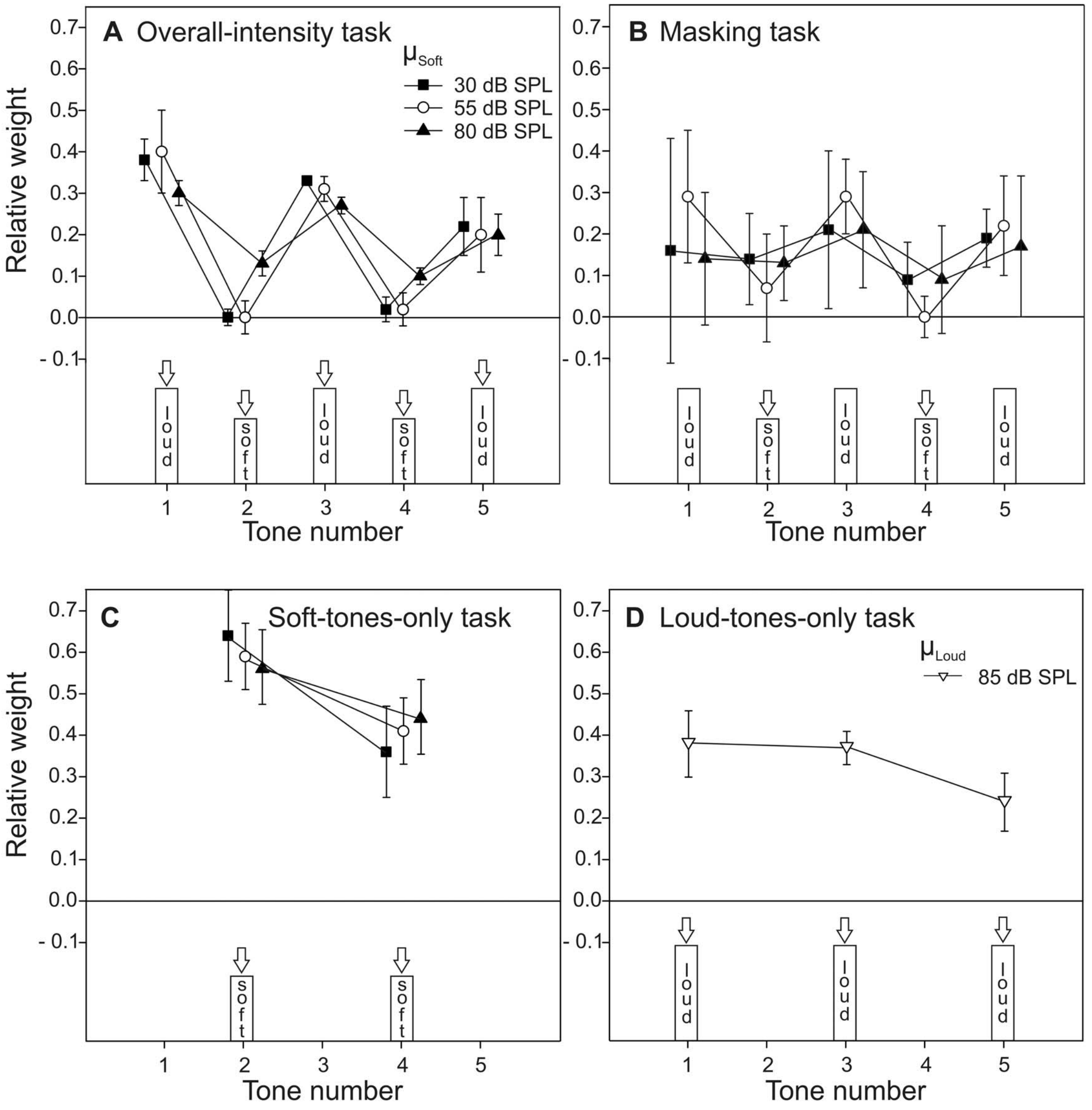

Figure 2. Mean normalized relative decision weights. Panel A: overall-intensity task. Panel B: masking task. Panel C: soft-tones-only task. Panel D: loud-tones-only task. Squares: $\mu_{soft}=30$ dB SPL. Circles: $\mu_{soft}=55$ dB SPL. Filled triangles: $\mu_{soft}=80$ dB SPL. Open triangles: $\mu_{loud}=85$ dB SPL. Arrows mark target tones containing the increment. Error bars represent 95%-CIs.
doi:10.1371/journal.pone.0079830.g002

no main effect of task. No significant tone number×task interaction was found, $F(6, 36)=1.52$, $p=.232$.

Sensitivity

Fig. 3 depicts the sensitivity in terms of d'_{2I} (converted from AUC; see Method section) for each combination of task and μ_{soft}. The sensitivity in the loud-tones-only task was slightly lower than in the soft-tones-only conditions. Three paired-samples *t*-tests (with correction for multiple testing) were computed to analyze the differences between the averaged d' in the loud-tones-only with the soft-tones-only task for $\mu_{soft}=30$, 55, and 80 dB SPL. For $\mu_{soft}=30$ and 55 dB SPL the differences were not significant. The sensitivity in the loud-tones-only task was significantly lower than in the 80 dB SPL soft-tones-only task. Thus, our use of different increments for the loud and the soft tones was successful in ensuring that the sensitivity for detecting the level increment on the soft tones was at least as high as for the loud tones. The sensitivity in the overall-intensity task was similar to the soft-tones-only task and increased with the mean level of the soft tones. For the masking task, the sensitivity was particularly low for the sequences containing the 55 dB SPL mean level of the soft tones, compatible with a mid-level hump pattern. In general, the sensitivity was low in this task ($d'<0.40$).

The sensitivity (d') was analyzed with an rmANOVA. The loud-tones-only task was excluded to provide a two-factorial design, task (overall-intensity, masking, soft-tones-only)×μ_{soft}. The effect of task was significant, $F(2, 12)=263.09$, $p<.001$, $\eta^2_p=.98$, confirming the large difference in d' between the masking task and the other two tasks. The effect of μ_{soft} was also significant, $F(2, 12)=26.82$, $p<.001$, $\eta^2_p=.82$. No significant Task×μ_{soft} interaction was found, $F(4, 24)=1.51$, $p=.232$.

To further analyze the effect of μ_{soft} on sensitivity, three separate rmANOVAs were computed. In the masking task, the data showed a mid-level hump pattern, although the effect of μ_{soft} was

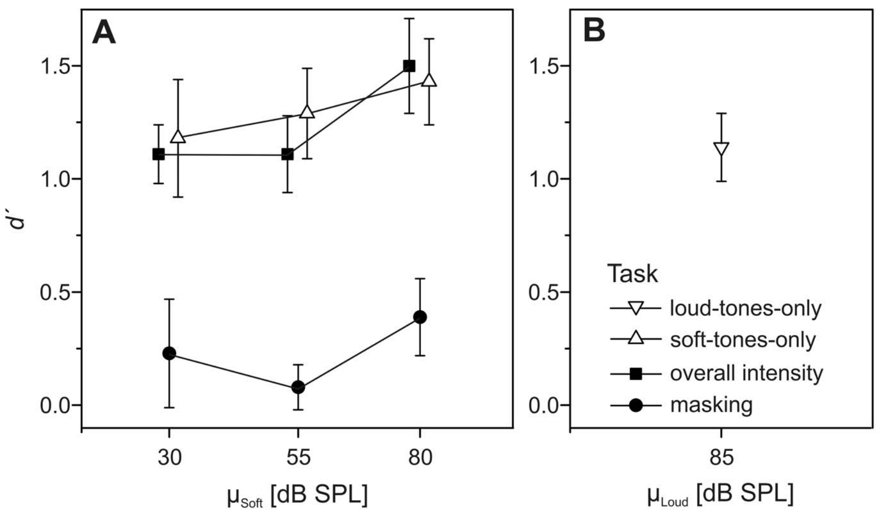

Figure 3. Average sensitivity (*d'*). Panel A: *d'* as a function of μ_{soft} and task. Squares: overall-intensity task. Circles: masking task. Triangles: soft-tones-only task. Panel B: *d'* in the loud-tones-only task. Error bars represent 95%-CIs.
doi:10.1371/journal.pone.0079830.g003

only marginally significant, $F(2, 12) = 3.69$, $p = .063$, $\eta^2_p = .38$, $\tilde{\varepsilon} = .91$. For the overall-intensity task the effect of μ_{soft} was significant, $F(2, 12) = 27.54$, $p < .001$, $\eta^2_p = .82$, $\tilde{\varepsilon} = .75$, but there was no evidence for a mid-level hump pattern of sensitivity. For the soft-tones-only task the effect of μ_{soft} was marginally significant, $F(2, 12) = 3.45$, $p = .066$, $\eta^2_p = .37$, and the higher sensitivity at higher levels was compatible with the near-miss to Weber's law.

Estimates of Internal Noise

Which factors limit observers' performance in the multitone intensity-discrimination task? As outlined in the Introduction, first, the information about intensity available at the decision stage might be inexact, for example because the loud tones reduce the precision of the information about the intensity of soft tones, which could be modeled as an increase in *internal noise* [14]. Second, it might be the case that a precise representation of tone intensity is available at the decision stage, but that this information is not used in an optimal fashion [14]. For example, in the masking task the decision might be influenced by task-irrelevant information about masker intensity (as observed by [23]). A reduction in sensitivity could be caused by either of these factors alone, or by a combination of both. Therefore, the sensitivity-based measures like the DL-elevation do not allow for deciding whether presentation of intense tones in a multitone sequence increases internal noise, or results in a suboptimal integration of information, or both. In contrast, with the "molecular psychophysics" methods used here, some insight can be gained into these two potential effects of non-simultaneous masking.

We used a maximum-likelihood approach to estimate the internal noise effective in the different experimental conditions. To illustrate this approach, imagine a sequence containing only two tones. On the basis of the type of decision variable assumed in Eq. (1), and compatible with the usual signal detection model, we assume the decision variable (i.e., value on the internal continuum) to be given by

$$X = w_1(L_1 + \varepsilon_1) + w_2(L_2 + \varepsilon_2), \quad (3)$$

where L_1 is the randomly varying sound pressure level of tone 1 on a given trial (including the level increment on increment-present trials), L_2 is the sound pressure level of tone 2, w_1 and w_2 is the decision weight assigned to the first and second tone, respectively, and ε_1 and ε_2 are random variables representing the internal noise effective for tone 1 and 2. The internal noise components ε_1 and ε_2 are assumed to be independent of each other and normally distributed with mean 0 and standard deviation σ_{I1} and σ_{I2}, respectively.

The probability of selecting a rating category $j = 1\ldots4$ (coded as 1 = "soft – rather sure", 2 = "soft – rather unsure", 3 = "loud – rather unsure", and 4 = "loud – rather sure") is

$$P(\text{response}=j) = \mathrm{CDF}[N(w_1L_1 + w_2L_2, \sqrt{w_1^2\sigma_{I1}^2 + w_2^2\sigma_{I2}^2}, c_j] - \mathrm{CDF}[N(w_1L_1 + w_2L_2, \sqrt{w_1^2\sigma_{I1}^2 + w_2^2\sigma_{I2}^2}, c_{j-1}] \quad (4)$$

where CDF[$N(\mu,\sigma),c$] is the cumulative density function of a normal distribution with mean μ and standard deviation σ, evaluated at the point c, $c_0 = -\infty$, and $c_4 = +\infty$.

This observer model can be used to obtain maximum likelihood estimates of the weights, and of the total internal noise standard deviation. For any pair of levels (L_1 and L_2), the likelihood of the observed rating response is given by Eq. (4). Assuming independence between trials, the total likelihood is the product of the likelihoods of the individual trials. We minimized the negative log likelihood numerically using the Mathematica 9.0 function *NMinimize*[]. The weights can only be estimated up to a multiplicative constant [6], which presents no problem because we are only interested in the relative weights. Therefore, without loss of generality we set $w_1 = 1$ or $w_1 = -1$, depending on whether the probability of selecting a higher rating category increases or decreases with the level of tone 1. As noted by Berg [6], the relative weights are independent of additive internal noise. In Eq. (4), the two internal noise variances only appear in a common term representing the standard deviation of the normally distributed decision variable. Therefore, increasing for example σ_{I1} will flatten out both "conditional on single stimulus" (COSS) [6] psychometric functions that describe the relation between for example L_1 and the probability of a given rating response, regardless of the level of the second tone (L_2). However, the

increase in σ_{I1} will not affect the *ratio* between the two estimated weights, w_1/w_2.

For the soft-tones-only condition, the internal noise can now be estimated from the trial-by-trial data by assuming $\sigma_{I1} = \sigma_{I2} = \sigma_{I\text{-soft}}$, i.e., that each stimulus component contributes the same amount of internal noise. For the loud-tones-only condition, a third term representing tone 3 is added to the equations (3) and (4), and it is assumed that $\sigma_{I1} = \sigma_{I2} = \sigma_{I3} = \sigma_{Iloud}$.

For the five-tone sequences (overall-intensity task and masking task) the mean and standard deviation of the cumulative-normal psychometric function relating the decision variable and the response are $\mu_{5T} = w_1\ L_1 + w_2\ L_2 + w_3\ L_3 + w_4\ L_4 + w_5\ L_5$ and $\sigma_{5T} = \sqrt{(w_1^2 + w_3^2 + w_5^2)\sigma_{Iloud5T}^2 + (w_2^2 + w_4^2)\sigma_{Isoft5T}^2}$, assuming that the internal noise does not differ between the three loud tones ($\sigma_{I1} = \sigma_{I3} = \sigma_{I5} = \sigma_{Iloud5T}$) or the two soft tones ($\sigma_{I2} = \sigma_{I3} = \sigma_{I\text{-soft5T}}$). The response probabilities are again given by

$$P(\text{response}=j) = \text{CDF}[N(\mu_{5T},\sigma_{5T}),c_j] - \text{CDF}[N(\mu_{5T},\sigma_{5T}),c_{j-1}]\ . \tag{5}$$

Unfortunately, it is not possible to obtain separate estimates of $\sigma_{Iloud5T}$ and $\sigma_{Isoft5T}$, because these parameters appear only in a common term. Yet, we can use the ML-estimate of σ_{5T} obtained from Eq. (5) for an approximate test of whether the internal noise standard deviation was increased in the five tone sequences, compared to sequences presenting only the loud or only the soft tones. To this end, we compare the estimate of σ_{5T} to $\sigma_{I\_isolated} = \sqrt{(w_1^2 + w_3^2 + w_5^2)\sigma_{Iloud}^2 + (w_2^2 + w_4^2)\sigma_{Isoft}^2}$, where w_1 through w_5 are the weights estimated in the five-tone sequence, and σ_{Isoft}^2 and σ_{Iloud}^2 is the internal noise variance estimated for the soft- and loud-tones-only condition, respectively. If we assume that combining the loud and soft tones into a single, alternating sequence can result in an increase, but not in a reduction of the internal noise effective for each of the two tones types (soft and loud), then if $\sigma_{5T} = \sigma_{I\_isolated}$ there was no increase in internal noise, while if $\sigma_{5T} > \sigma_{I\_isolated}$ then at least one of the internal noises was increased in the alternating sequence. This rationale corresponds to the observer efficiency [62] measure η_{noise} proposed by Berg [8]. The latter measure is defined as $\eta_{noise} = (d'_{obs}/d'_{wgt})^2$, where d'_{wgt} is the sensitivity that would result if the internal noises in the five-tone sequence were identical to the internal noises in the sequences containing only soft or only loud tones, and if the listener applied the decision weights actually observed for the five-tone task. If the observed sensitivity in the five tone task, d'_{obs}, is smaller than d'_{wgt} (i.e., $\eta_{noise} < 1$), then there was an increase in internal noise.For large samples (i.e., high number of data points, as in the present experiment), maximum likelihood estimates are approximately normal [63], and their asymptotic variance-covariance matrix can be calculated in terms of the Fisher information, by taking the inverse of the Hessian matrix [63]. It is therefore possible to test H_0: $\sigma_{5T} = \sigma_{I\_isolated}$ on an individual basis. The ML analysis of the data from the five-tone sequences provides estimates of the weights, their standard errors (SE), and their covariances, as well as an estimate of σ_{5T} and its SE. ML analyses of the data from the soft−/loud-tones-only conditions provide estimates and SEs of σ_{Iloud} and σ_{Isoft}. We were not able to find an analytic solution for the standard error of $\sigma_{I\_isolated}$. We therefore used a Monte Carlo approach, where we simulated 200,000 values of $\sigma_{I\_isolated}$, with w_1 through w_5, σ_{Isoft}, and σ_{Isoft} drawn from normal distributions, with means, standard deviations and covariances as estimated via the ML analyses. The mean and standard deviation of the simulated samples of $\sigma_{I\_isolated}$ were taken as estimates of the population mean and the standard deviation of $\sigma_{I\_isolated}$. The simulated weights were multinormally distributed, with mean vector $\{w_1, w_2, w_3, w_4, w_5\}$ and covariance matrix as estimated by the ML analysis of the five-tone conditions. The simulated "isolated" internal noises σ_{Isoft} and σ_{Isoft} were independent and normally distributed with means and standard deviations as estimated by the ML analysis of the soft−/loud-tones-only conditions. For a given subject and condition, the test statistic $z_{diff} = (\hat{\sigma}_{I\_isolated} - \hat{\sigma}_{5T})/SE_{diff}$, where $SE_{diff} = \sqrt{SE_{\sigma_{I\_isolated}}^2 + SE_{\sigma_{5T}}^2}$, can be referred to a standard normal distribution, providing a test of H_0: $\sigma_{I\_isolated} = \sigma_{5T}$ against H_1: $\sigma_{I\_isolated} \neq \sigma_{5T}$.

The relative weights obtained by the described maximum likelihood analysis were almost identical to the relative weights provided by the multiple regression analyses presented in section *Decision weights* ($R^2 = .99$), compatible with previous results [64,65] and confirming the validity of our analyses. The individual estimates of σ_{5T} and $\sigma_{I\_isolated}$ for the overall-intensity task are displayed in Fig. 4. At an α-level of.05 (two-tailed), there was no significant difference between σ_{5T} and $\sigma_{I\_isolated}$, except for one case where σ_{5T} was even lower than $\sigma_{I\_isolated}$. Thus, the data indicate that the combination of the loud and soft tones into an alternating sequence did not increase the internal noise.

Given that there was no evidence for an increase in internal noise, it is interesting to quantify the loss in sensitivity caused by non-optimal decision weights, in particular the low weights on the soft tones. To this end, we estimated the sensitivity that a "limited ideal observer" [66] would obtain using the optimal set of decision weights in the overall-intensity task in the absence of any masking effects (i.e., no increase in internal noise compared to sequences containing only loud or only soft tones, as demonstrated by the above analysis). By definition, d' is equal to the difference between the expected value of the decision variable on increment-present and increment-absent trials, divided by the common standard deviation [36]. For example, in the two-tones sequences considered above in Eq. (3), the decision variable on increment-present trials is given by

$$X_{incr} = w_1\ (L_{1P} + \Delta L_1 + \varepsilon_1) + w_2(L_{2P} + \Delta L_2 + \varepsilon_2)\ , \tag{6}$$

where L_{1P} is the randomly varying "pedestal" level of the first tone presented on the given trial, ΔL_1 is the level increment imposed on the first tone, ε_1 is a random variable representing internal noise, and w_1 is the decision weight assigned to the first tone. The second term on the right side represents the same parameters for the second tone. Due to the random level perturbations (representing "external noise"), L_{1P} and L_{2P} are independent random variables with mean μ_1 and μ_2, respectively, and standard deviation $\sigma_{E1} = \sigma_{E2} = \sqrt{1/12 \cdot q^2}$, where $q = 12$ dB is the range of the uniform distribution L_{1P} and L_{2P} were sampled from. As a reminder, the internal noise components ε_1 and ε_2 are assumed to be independent of each other and independent of the external noise, normally distributed with mean 0 and standard deviation σ_{I1} and σ_{I2}, respectively.

For the increment-absent trials, the decision variable corresponds to

$$X_{noIncr} = w_1\ (L_{1P} + \varepsilon_1) + w_2(L_{2P} + \varepsilon_2)\ . \tag{7}$$

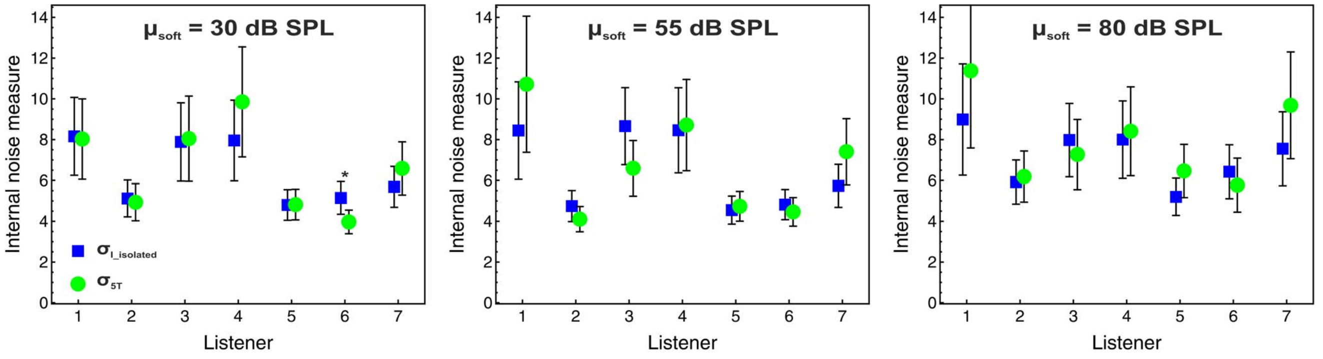

Figure 4. Individual internal noise estimates for the overall-intensity task. The panels represent the mean levels of the soft tones. Blue squares: $\sigma_{I\_isolated}$. Green circles: σ_{5T}. Error bars represent 95%-CIs. The asterisk indicates a significant difference between σ_{5T} and $\sigma_{I\_isolated}$ ($p<.05$).
doi:10.1371/journal.pone.0079830.g004

By definition, d' is equal to the mean of $X_{incr} - X_{noIncr}$ divided by the common standard deviation. Thus,

$$d' = \frac{E(X_{incr} - X_{noIncr})}{\sqrt{E\left(X^2_{incr}\right) - E^2(X_{incr})}}. \tag{8}$$

For our setting, the general formula for $i = 1 \ldots k$ loud and $j = 1 \ldots l$ soft tones is

$$d' = \frac{\Delta L_L \sum W_{Li} + \Delta L_S \sum w_{Sj}}{\sqrt{\sum w^2_{Li}\left(q^2/12 + \sigma^2_{ILoud}\right) + \sum w^2_{Sj}\left(q^2/12 + \sigma^2_{ISoft}\right)}}, \tag{9}$$

where Σw_{Li} and Σw_{Sj} denotes the sum of the weights assigned to the k loud and l soft tones, respectively, and ΔL_L and ΔL_S are the level increments imposed on the two types of tones. On the basis of the internal noise variances estimated from the data obtained in the soft- and loud-tones-only conditions, we computed the sensitivity d'_{OWNM}, where the index stands for 'optimal weights, no masking'. This is the sensitivity that would result for the five-tone sequences if (a) there was no increase in internal noise compared to the soft-tones-only or loud-tones-only condition, and (b) the listener applied the optimal decision weights. In the overall-intensity task, the listener can use information from five tones. These five observations can be assumed to be independent, because the "external noise" (level variation) applied to each tone was independent, we assume the internal noise components to be mutually independent and independent of the external noise, and we assume that there are no masking effects. Therefore, if the listener optimally combines the information from the five tones (integration model; [16]), then the sensitivity is

$$d'_{OWNM} = \sqrt{\sum_{i=1}^{5} (d'_i)^2} = \sqrt{\sum_{i=1}^{5}\left(\frac{\Delta L_i}{\sqrt{q^2/12 + \sigma^2_{Ii}}}\right)^2}, \tag{10}$$

where d'_i is the sensitivity for tone i, which is given by the difference between increment-present and increment-absent trials divided by the standard deviation of this difference. The SD is a function of the external noise ($\sigma_E = \sqrt{q^2/12}$) and the internal noise (σ_{Ii}). Because $\Delta L_i = \Delta L_L$ and $\sigma_{Ii} = \sigma_{ILoud}$ for tones 1, 3 and 5, and $\Delta L_i = \Delta L_S$ and $\sigma_{Ii} = \sigma_{ISoft}$ for tones 2 and 4,

$$d'_{OWNM} = \sqrt{\left(\frac{\sqrt{3}\Delta L_L}{\sqrt{q^2/12 + \sigma^2_{ILoud}}}\right)^2 + \left(\frac{\sqrt{2}\Delta L_S}{\sqrt{q^2/12 + \sigma^2_{ISoft}}}\right)^2}. \tag{11}$$

The first term under the root is the d' that can be achieved using information from only the loud tones, and the second term is d' when using only the soft tones. This optimal sensitivity is obtained if each loud tone receives identical weight ($w_1 = w_3 = w_5 = w_L$), each soft tone receives identical weight ($w_2 = w_4 = w_S$), and.

$$w_S = \frac{\Delta L_S(q^2/12 + \sigma^2_{IL})}{\Delta L_L(q^2/12 + \sigma^2_{IS})} w_L. \tag{12}$$

The interpretation of Eq. (12) is simple: the optimum ratio between the weight assigned to the soft tones and the weight assigned to the loud tones is the reliability (level increment divided by the sum of the internal and external noise variance) of the soft tones, divided by the reliability of the loud tones.

Table 1. Mean sensitivities and efficiency estimates depending on the task and the level of the soft tones.

	μ_{soft} (dB SPL)	d'_{obs}	d'_{OWNM}	$\eta = (d'_{obs}/d'_{OWNM})^2$
Overall-intensity task	30	1.11 (0.14)	1.77 (0.22)	0.41* (0.12)
	55	1.11 (0.18)	1.83 (0.17)	0.37* (0.10)
	80	1.50 (0.23)	2.02 (0.23)	0.55* (0.11)
Masking task	30	0.23 (0.26)	1.22 (0.24)	0.05* (0.08)
	55	0.08 (0.11)	1.31 (0.20)	0.01* (0.02)
	80	0.39 (0.18)	1.57 (0.20)	0.08* (0.03)

Note. Values in brackets represent the standard deviation of averaged values across listeners. *: η significantly different from 1.0 (*t*-test for one sample, two-tailed, $p<.05$).
doi:10.1371/journal.pone.0079830.t001

The average values of d'_{OWNM} are displayed in Table 1, together with the observed sensitivity d'_{obs}. The observer efficiency can be defined as $\eta = (d'_{\text{obs}}/d'_{\text{OWNM}})^2$ [8,62]. The important difference between our analysis and the efficiency measures proposed by Berg [8] is that he used the sensitivity for an ideal observer with zero internal noise and ideal weights (termed d'_{ideal}) as the reference sensitivity relative to which the observer efficiency was computed. Our reference sensitivity, d'_{OWNM}, differs from this concept because it encompasses the internal noise present in the soft−/loud-tones-only condition. We selected this reference sensitivity because we were interested in changes in internal noise in the alternating-level five-tone sequences relative to the conditions presenting only the soft or only the loud tones, rather than in a comparison to an ideal observer characterized by the (unrealistic) complete absence of internal noise. A similar analysis was used by Alexander and Lutfi [66], who for a multitone frequency discrimination task computed the reference sensitivity on the basis of the frequency resolution for individual frequency components and termed this reference sensitivity d'_{PLIO} for "peripherally limited ideal observer".

Because in the overall-intensity task there was no evidence for increased internal noise, values of η lower than 1.0 represent a loss in sensitivity due to non-optimal weights, and for the overall-intensity task η was significantly smaller than 1.0 at all mean levels of the soft tones. Thus, in the overall-intensity task the internal noise was not higher than for the soft tones or loud tones presented alone, but the listeners did not achieve the maximally possible sensitivity because they used suboptimal decision weights. The efficiency was higher at $\mu_{\text{soft}} = 80$ dB SPL than at the two lower levels of the soft tones. An rmANOVA on η showed a significant effect of μ_s, $F(2,12) = 4.85$, $p = .035$, $\eta^2_p = .45$, $\tilde{\varepsilon} = .88$. This pattern is similar to the observed sensitivity (see Fig. 3).

For the masking task, the individual estimates of σ_{5T} and $\sigma_{I\_isolated}$ are displayed in Fig. 5. The confidence intervals show that in several cases it was not possible to obtain precise ML-estimates of σ_{5T}. In one case (listener 6 at $\mu_s = 55$ dB SPL) no acceptable model fit could be obtained. Unlike in the overall-intensity task, σ_{5T} tended to be higher than $\sigma_{I\_isolated}$. The difference between the two internal noise measures was significant ($p < .05$) for four out of six listeners at the lowest, three listeners at the intermediate and one listener at the highest mean level of the soft tones. Thus, for the masking task an increase in internal noise in the five-tone sequences cannot be precluded. Because it is not possible to separately estimate σ_{Iloud5T} and σ_{Isoft5T}, the increase in internal noise could be due to a reduced precision of the intensity information for both the loud and soft tones, although it seems likely that the presence of the loud tones increased the internal noise effective for the soft tones due to non-simultaneous masking [11]. Apart from the evidence for increased internal noise, the non-zero weights observed on the loud tones in the masking task (see Fig. 2) clearly represent non-optimal weights because no level increment was presented on the loud tones, so that they conveyed no information concerning the correct response. However, it is unfortunately not possible to use the *weighting efficiency* $\eta_{\text{wgt}} = (d'_{\text{wgt}}/d'_{\text{OWNM}})^2$ that Berg [8] proposed as a measure of the loss in efficiency caused by suboptimal weights. The measure d'_{wgt} is defined as the sensitivity that would result if (a) the listener applied the decision weights actually observed for the masking task and if (b) there was no increase in internal noise. Because unlike for the overall-intensity task we cannot rule out an increase in internal noise, separate estimates of σ_{Iloud5T} and σ_{Isoft5T} would again be necessary for quantifying the additional loss in sensitivity in the masking task caused by non-optimal weights. As shown in Table 1, the observer efficiency (η) in the masking task was very low and significantly smaller than 1.0 for all values of μ_{soft}.

Discussion

The present experiment studied the relation between forward-masked intensity discrimination and multitone intensity discrimination. Previous data showed a strong impairment in intensity resolution caused by non-simultaneous maskers (for a review see [11]), and a systematic bias towards low decision weights assigned to soft tones in a multitone intensity-discrimination task presenting an alternating sequence of loud and soft tones [7]. To investigate the relationship between the two tasks we presented a five-tone sequence containing alternating loud and soft tones. The level of the loud tones was fixed at 85 dB SPL, and the level of the soft tones was varied (30, 55, and 80 dB SPL), corresponding to the level combinations representing the mid-level hump in forward-masked intensity discrimination. The task was to detect a level increment on either all tones of the multitone stimulus (overall-intensity task), or only on the soft tones while ignoring the loud tones (masking task). The latter condition should allow for a comparison with a typical forward-masked intensity-discrimination task.

As expected, listeners did not assign uniform temporal weights. In the overall-intensity task, the weights on the loud tones exhibited a primacy effect, i.e., a higher weight was assigned to the first tone than to the following loud tones. The same pattern was

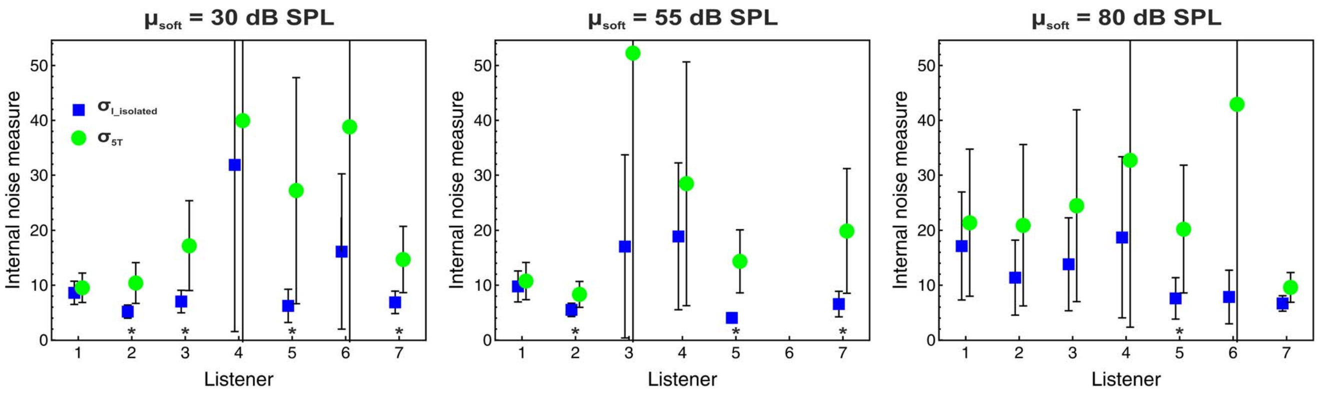

Figure 5. Individual internal noise estimates for the masking task. Same format as Fig. 4. At $\mu_s = 55$ dB SPL, no acceptable fit of the model could be obtained for listener 6.
doi:10.1371/journal.pone.0079830.g005

present in the soft−/loud-tones-only conditions. This is compatible with previous reports of temporal weights in intensity-discrimination tasks [5,28,59,60,61].

Concerning the decision strategy in the sequences containing both soft and loud tones, we expected two main results. First, in the overall-intensity task we anticipated smaller weights on soft than on loud tones. In the masking task, we expected non-zero weights on the to-be-ignored loud tones, and thus a systematic influence of the uninformative masker-intensities on the decision [23]. Additionally, these effects were expected to be particularly strong at the intermediate level of the soft tones (55 dB SPL). This hypothesis is compatible with the conception that the difficulty in selectively attending to the (soft) target tones in a classical forward-masked intensity-discrimination task is a major factor for the deterioration of intensity resolution [22,23,24], which is particularly strong at mid-levels. Second, we were interested in whether listeners would only show non-optimal decision weights or if there would be evidence for an increase in internal noise in the five-tone sequences.

As expected, the weights assigned to the soft tones were lower than the weights assigned to the loud tones in the overall-intensity task. The weights on the soft tones were similar and near zero for $\mu_{soft} = 30$ and 55 dB SPL, and significantly higher at $\mu_{soft} = 80$ dB SPL, compatible with results by Lutfi and Jesteadt [7]. Thus, the weighting patterns did not exhibit a clear mid-level hump like pattern. In the masking task, the loud tones (maskers) received non-zero weights, confirming our expectation that listeners would fail to ignore the maskers. This pattern was most pronounced at the intermediate level of the soft tones, where the weights on the soft tones (targets) were even lower than the weights on the loud tones (maskers), compatible with a mid-level hump. The difference between the weighting patterns observed at the three levels of the soft tones was not significant, however. It should be noted that in the masking task there was also only weak evidence for lower sensitivity at the intermediate than at the low or high target level. Thus, in terms of intensity resolution, the mid-level hump pattern was weaker than we had expected.

What can be concluded about the origin of the reduced sensitivity in the alternating-level sequences? We first discuss the overall-intensity task, where level increments were presented on all of the five tones. Despite the fact that the higher level increments presented on the soft tones slightly overcompensated for the near-miss to Weber's law (see section *Sensitivity*), so that the soft tones provided at least the same amount of information as the loud tones, at the two lower soft-tone levels only the loud tones received considerable decision weights while the soft tones were virtually ignored. Regarding the decision about the intensity of the complete sequence, the listener would make optimal use of available information by using the level information from *all* of the five tones. However, as Figure 3 shows, almost ignoring the information provided by the soft tones still resulted in an acceptable level of performance. Thus, one could argue that the listeners might have adopted an "economical" strategy by attending only to the loud tones. Alternatively, it might have been the case that the loud tones acted as forward maskers, rendering the information about the level of the soft tones available at the decision stage imprecise (in the sense of increased internal noise), and thus uninformative. The molecular analyses providing separate estimates of decision weights and internal noise allow rejection of the latter hypothesis, however. In the overall-intensity task we found no evidence for an increase in total internal noise compared to the loud- or soft-tones-only conditions. Therefore, the observed weighting pattern in the overall-intensity task cannot be attributed to impaired information about soft tone intensity. Instead, the data are compatible with the concept that the information about the intensity of the soft tones is not used optimally due to a failure of selective attention to the soft tones (cf. [11,22,23,24]), as evidenced by the estimates of observer efficiency (Table 1). Our data are also compatible with the observation of effects of relative tone level at long ISIs in sample discrimination of frequency [9] where "masking" effects are very unlikely, and therefore the higher reliance on information from the louder stimulus components can also be viewed as a suboptimal weighting strategy.

For the masking task where only the soft tones provided information concerning the correct response, the relative weights on the soft tones were higher than in the overall-intensity task, except at the intermediate target level (55 dB SPL). Thus, the listeners were in principle able to adjust their decision weights based on the task requirements. However, the decision was systematically influenced by the uninformative intensity of the loud tones. This result is directly compatible with the hypothesis that a failure of selective attention to the targets in an intensity-discrimination task under non-simultaneous masking is a major factor in the sometimes dramatic reduction in intensity resolution [22,23,24]. In a classical intensity-discrimination task with only one target and one forward-masker, Oberfeld [23] also found that the masker intensity was factored into the decisions. For the masking task, our data indicated an increase in internal noise for some listeners and levels of the soft tones. Why could the combination of the soft and loud tones into an alternating sequence cause an increase in internal noise in the masking task but not in the overall-intensity task? This result is quite unexpected because the temporal sequence of loud and soft tones was virtually identical in the two tasks (apart from the 4 dB level increment placed on the loud tones in the overall-intensity task but not in the masking task). For this reason, any adaptation effects in the auditory periphery caused by the loud tones should have been identical in the two tasks. Put differently, because the two tasks presented virtually the same stimuli but differed in their task requirements (attend to all tones in the overall-intensity task, attend only to the soft tones in the masking task), the increase in internal noise appears to be caused by higher-level processes rather than by peripheral mechanisms. More specifically, our analyses suggest that in the masking task the listeners did not only apply suboptimal weighting strategies, but that there was an additional higher level process causing an increase in internal noise. Additional research is necessary to identify this process.

In our observer model (cf. Eq. (3)), the internal noise components associated with each tone are modeled as being 'early', that is, they appear prior to integration. As a consequence, the influence of the internal noise on the decision variable is modulated by the decision weight. If the weight assigned to a given tone is 0, then the internal noise associated with this tone will not contribute to the effective total internal noise. An internal noise source located at or after integration would have a different effect because its contribution is not modulated by the decision weights. Such an additional 'central' internal noise source (e.g., [67]) could be integrated into an extended version of the model. In the present study, we wanted to answer the question of whether the loud tones impair the representation of soft-tone intensity, modeled as an increase in 'early', 'sensory' noise. An additional 'central' noise source would equally affect the information available from the soft tones and the loud tones, and could therefore neither explain the relatively low decision weights assigned to the soft tones in the overall-intensity task, nor the non-zero weights assigned to the loud "maskers" in the masking task. More specifically, if the central noise dominated the 'sensory' noise, then the listeners

should assign approximately uniform weights, regardless of the internal noise SDs effective for the soft versus the loud tones. This is because in Eq. (12) specifying the optimal ratio between the decision weights for the loud and soft tones, the central noise variance would appear in both the numerator and the denominator. Moreover, the additional noise source would not affect the estimates of relative decision weights, for reasons discussed in Section Estimates of internal noise. However, an increased level of central internal noise in the five-tone sequences compared to the soft-tones-only or loud-tones-only conditions might partly explain the observation of increased total internal noise in some conditions. Data by Lutfi and Gilbertson [68] are evidence against a significant contribution of central noise in a sample discrimination task. However, as they studied sample discrimination of frequency rather than intensity, it remains to be shown if such evidence can be obtained for the task we studied.

Apart from the insights into multitone intensity discrimination, we present a new analysis method for obtaining maximum likelihood estimates of decision weights and internal noise. This method has advantages over the observer efficiency analysis used in previous studies [8,69] because it does not refer to an ideal observer characterized by the complete absence of internal noise. It would be interesting to apply the same type of molecular analyses to a classical intensity discrimination task instead of the five-tone task studied in the present paper. The method can also be adopted to study other tasks or other sensory modalities. For example, in an experiment on object-based visual attention (cf. [70,71]), the methods described in this paper could be used to quantify the amount of selective attention to the target in the different conditions by measuring the decision weights assigned to target and distractor elements, and a potential increase in internal noise caused by the distractor elements could be identified.

Acknowledgments

We are grateful to Wolfgang Ellermeier and an anonymous reviewer for helpful comments on a previous version of this paper. We thank Franziska Pfeffer and Felicitas Klöckner for their assistance in manuscript preparation.

Author Contributions

Conceived and designed the experiments: DO MK WJ. Performed the experiments: DO MK. Analyzed the data: DO MK. Contributed reagents/materials/analysis tools: DO MK. Wrote the paper: DO MK WJ.

References

1. Lutfi RA (1989) Informational processing of complex sound. I: Intensity discrimination. J Acoust Soc Am 86: 934–944.
2. Oberfeld D (2008) Does a rhythmic context have an effect on perceptual weights in auditory intensity processing? Canadian Journal of Experimental Psychology/Revue canadienne de psychologie experimentale 62: 24–32.
3. Green DM (1964) Consistency of auditory detection judgments. Psychol Rev 71: 392–407.
4. Ahumada AJ, Lovell J (1971) Stimulus features in signal detection. J Acoust Soc Am 49: 1751–1756.
5. Oberfeld D, Plank T (2011) The temporal weighting of loudness: Effects of the level profile. Attention, Perception & Psychophysics 73: 189–208.
6. Berg BG (1989) Analysis of weights in multiple observation tasks. J Acoust Soc Am 86: 1743–1746.
7. Lutfi RA, Jesteadt W (2006) Molecular analysis of the effect of relative tone level on multitone pattern discrimination. J Acoust Soc Am 120: 3853–3860.
8. Berg BG (1990) Observer efficiency and weights in a multiple observation task. J Acoust Soc Am 88: 149–158.
9. Turner MD, Berg BG (2007) Temporal limits of level dominance in a sample discrimination task. J Acoust Soc Am 121: 1848–1851.
10. McGill WJ, Goldberg JP (1968) A Study of the Near-Miss Involving Webers Law and Pure-Tone Intensity Discrimination. Percept Psychophys 4: 105–109.
11. Oberfeld D (2008) The mid-difference hump in forward-masked intensity discrimination. J Acoust Soc Am 123: 1571–1581.
12. Zeng FG, Turner CW (1992) Intensity discrimination in forward masking. J Acoust Soc Am 92: 782–787.
13. Zeng FG, Turner CW, Relkin EM (1991) Recovery from prior stimulation II: Effects upon intensity discrimination. Hear Res 55: 223–230.
14. Swets JA, Shipley EF, McKey MJ, Green DM (1959) Multiple observations of signals in noise. J Acoust Soc Am 31: 514–521.
15. Durlach NI, Braida LD (1969) Intensity perception: I. Preliminary theory of intensity resolution. J Acoust Soc Am 46: 372–383.
16. Green DM (1958) Detection of multiple component signals in noise. J Acoust Soc Am 30: 904–911.
17. Leshowitz B, Cudahy E (1973) Frequency discrimination in the presence of another tone. J Acoust Soc Am 54: 882–887.
18. Turner CW, Zeng FG, Relkin EM, Horwitz AR (1992) Frequency discrimination in forward and backward masking. J Acoust Soc Am 92: 3102–3108.
19. Massaro DW (1975) Backward recognition masking. J Acoust Soc Am 58: 1059–1065.
20. Plack CJ, Carlyon RP, Viemeister NF (1995) Intensity discrimination under forward and backward masking: role of referential coding. J Acoust Soc Am 97: 1141–1149.
21. Plack CJ, Viemeister NF (1992) Intensity discrimination under backward masking. J Acoust Soc Am 92: 3097–3101.
22. Oberfeld D, Stahn P (2012) Sequential grouping modulates the effect of non-simultaneous masking on auditory intensity resolution. PLoS ONE 7: e48054.
23. Oberfeld D (2009) The decision process in forward-masked intensity discrimination: Evidence from molecular analyses. J Acoust Soc Am 125: 294–303.
24. Oberfeld D, Stahn P, Kuta M (2012) Binaural release from masking in forward-masked intensity discrimination: Evidence for effects of selective attention. Hear Res 284: 1–9.
25. Oberfeld D (2010) Electrophysiological correlates of intensity resolution under forward masking. In: Lopez-Poveda EA, Palmer AR, Meddis R, editors. Advances in Auditory Research: Physiology, Psychophysics and Models. New York: Springer. 99–110.
26. Braida LD, Durlach NI (1972) Intensity perception: II. Resolution in one-interval paradigms. J Acoust Soc Am 51: 483–502.
27. Alexander JM, Lutfi RA (2004) Informational masking in hearing-impaired and normal-hearing listeners: Sensation level and decision weights. J Acoust Soc Am 116: 2234–2247.
28. Pedersen B, Ellermeier W (2008) Temporal weights in the level discrimination of time-varying sounds. J Acoust Soc Am 123: 963–972.
29. Békésy Gv (1947) A new audiometer. Acta Otolaryngol (Stockh) 35: 411–422.
30. Hartmann WM (2005) Signals, Sound, and Sensation: Springer.
31. Zwillinger D, editor (2003) CRC Standard Mathematical Tables and Formulae. 31st ed. Boca Raton: CRC.
32. IEC 318 (1970) An IEC artificial ear, of the wide band type, for the calibration of earphones used in audiometry. Geneva: International Electrotechnical Commission.
33. Nachmias J (2006) The role of virtual standards in visual discrimination. Vision Res 46: 2456–2464.
34. Berg BG, Robinson DE (1987) Multiple Observations and Internal Noise. J Acoust Soc Am 81: S33–S33.
35. Sorkin RD, Robinson DE, Berg BG (1987) A detection theory method for the analysis of auditory and visual displays. Proceedings of the 31st Annual Meeting of the Human Factors Society. 1184–1188.
36. Green DM, Swets JA (1966) Signal detection theory and psychophysics. New York: Wiley.
37. Swets JA (1986) Form of empirical ROCs in discrimination and diagnostic tasks: implications for theory and measurement of performance. Psychol Bull 99: 181–198.
38. Swets JA (1986) Indices of discrimination or diagnostic accuracy: their ROCs and implied models. Psychol Bull 99: 100–117.
39. Macmillan NA, Rotello CM, Miller JO (2004) The sampling distributions of Gaussian ROC statistics. Percept Psychophys 66: 406–421.
40. Iverson G, Bamber D (1997) The generalized area theorem in signal detection theory. In: Luce RD, Marley AAJ, editors. Choice, decision, and measurement: essays in honor of R Duncan Luce. Mahwah, N.J.: L. Erlbaum. pp. xv, 472 p.
41. Green DM, Moses FL (1966) On the Equivalence of two Recognition Measures of Short-Term Memory. Psychol Bull 66: 228–234.
42. Hanley JA, McNeil BJ (1982) The Meaning and Use of the Area under a Receiver Operating Characteristic (ROC) Curve. Radiology 143: 29–36.
43. Macmillan NA, Creelman CD (2005) Detection Theory: A User's Guide. Mahwah, NJ: Lawrence Erlbaum Associates. XIX, 492 S. p.
44. Buus S, Florentine M (1991) Psychometric functions for level discrimination. J Acoust Soc Am 90: 1371–1380.
45. Moore BCJ, Peters RW, Glasberg BR (1999) Effects of frequency and duration on psychometric functions for detection of increments and decrements in sinusoids in noise. J Acoust Soc Am 106: 3539–3552.
46. Oberfeld D, Franke T (2013) Evaluating the robustness of repeated measures analyses: The case of small sample sizes and non-normal data. Behavior Research Methods 45: 792–812.
47. Agresti A (2002) Categorical data analysis. New York, NY: Wiley. XV, 710 S. p.

48. Agresti A (1989) Tutorial on modeling ordered categorical response data. Psychol Bull 105: 290–301.
49. McCullagh P (1980) Regression models for ordinal data. Journal of the Royal Statistical Society Series B: Methodological 42: 109–142.
50. Levitt H (1971) Transformed up-down methods in psychoacoustics. J Acoust Soc Am 49: Suppl 2: 467–477.
51. Green DM, von Gierke SM, Hanna TE (1986) Absolute intensity discrimination using two responses. J Acoust Soc Am 79: 760–766.
52. Cohen J (1988) Statistical power analysis for the behavioral sciences. Hillsdale, N.J.: L. Erlbaum Associates.
53. Huynh H, Feldt LS (1976) Estimation of the Box correction for degrees of freedom from sample data in randomized block and split-plot designs. Journal of Educational Statistics 1: 69–82.
54. Maxwell SE, Delaney HD (2004) Designing experiments and analyzing data: A model comparison perspective. Mahwah, N.J.: Lawrence Erlbaum Associates. 1 v. (various pagings) p.
55. Keselman HJ (1994) Stepwise and simultaneous multiple comparison procedures of repeated measures' means. Journal of Educational Statistics 19: 127–162.
56. Hochberg Y (1988) A sharper Bonferroni procedure for multiple tests of significance. Biometrika 75: 800–802.
57. Baddeley AD (1966) Short-term memory for word sequences as a function of acoustic semantic and formal similarity. Q J Exp Psychol 18: 362–365.
58. Ellermeier W, Schrödl S (2000) Temporal weights in loudness summation. In: Bonnet C, editor. Fechner Day 2000 Proceedings of the 16th Annual Meeting of the International Society for Psychophysics. Strasbourg: Université Louis Pasteur. 169–173.
59. Rennies J, Verhey JL (2009) Temporal weighting in loudness of broadband and narrowband signals. J Acoust Soc Am 126: 951–954.
60. Dittrich K, Oberfeld D (2009) A comparison of the temporal weighting of annoyance and loudness. J Acoust Soc Am 126: 3168–3178.
61. Oberfeld D, Heeren W, Rennies J, Verhey JL (2012) Spectro-temporal weighting of loudness. PLoS ONE 7: e50184.
62. Tanner WP, Birdsall TG (1958) Definitions of d' and eta as psychophysical measures. J Acoust Soc Am 30: 922–928.
63. Cramér H (1946) Mathematical methods of statistics. Princeton: Princeton University Press.
64. Tang Z, Richards VM, Shih A (2005) Comparing linear regression models applied to psychophysical data. The Journal of the Acoustical Society of America 117: 2597.
65. Plank T (2005) Auditive Unterscheidung von zeitlichen Lautheitsprofilen (Auditory discrimination of temporal loudness profiles) [PhD thesis]. Regensburg: Universität Regensburg.
66. Alexander JM, Lutfi RA (2008) Sample discrimination of frequency by hearing-impaired and normal-hearing listeners. J Acoust Soc Am 123: 241–253.
67. Durlach NI, Braida LD, Ito Y (1986) Towards a model for discrimination of broadband signals. J Acoust Soc Am 80: 63–72.
68. Lutfi RA, Gilbertson L (2011) Effect of decision weights and internal noise on the growth of d' with N. J Acoust Soc Am 130: EL329–EL333.
69. Berg BG (2004) A molecular description of profile analysis: Decision weights and internal noise. J Acoust Soc Am 115: 822–829.
70. Chen Z (2012) Object-based attention: A tutorial review. Attention, Perception, & Psychophysics 74: 784–802.
71. Kahneman D, Henik A (1981) Perceptual organization and attention. In: Kubovy M, Pomerantz JR, editors. Perceptual Organization. Hillsdale, NJ: Erlbaum. 181–211.
